# The Education of the Dental Surgeon

**Published:** 1899-02

**Authors:** Frank Woodbury

**Affiliations:** Halifax, N. S.


### AMERICAN JOURNAL

—OF—

### DENTAL SCIENCE.

VOL. XXXII. BALTIMORE, FEBRUARY, 1899. NO. 10.

### Selections.

ARTICLE I.

### THE EDUCATION OF THE DENTAL SURGEON.

FRANK WOODBURY, D. D. S.. HALIFAX, N. S.

Read before the Maritime Dental Association, Sept. 1898.

I am aware that many differ widely from some of the opinions contained in this short paper.

All will, however, agree at the outset that, everything else being equal, *the man of highest culture is the best man.* It matters not whether he has acquired it at the university halls, or by economy of his spare moments, the scale turns for the man of actual culture.

Before the days of dental boards and dental laws every man was a law to himself. The standard of preparation for his life-work was left to convenience, taste, or, perchance, force of circumstances.

We are building to-day on the foundations laid by our fathers in the profession, and we may fittingly make obeisance to them for the magnificent things they accomplished for us; but notwithstanding all that, I believe we are building on a foundation that never should have been laid.

I grant you that they moved from the blacksmith and barbar shop to the office—transformed the tooth-puller into the dental surgeon, and provided colleges where the science and art of dentistry are systematically taught.

It was an immense stride, but, from our point of view we cannot help but regret that the stride was in the wrong direction. It was a mistake. It was out of harmony with the fitness of things.

A preliminary requirement should have been *a course in a medical college.* Dentistry is a specialty in medicine *by right.*

The contributions that dentistry has made to medical and surgical science are not a few, and there is no reason why it should not stand shoulder to shoulder with other specialties, and immeasurably better than it does, except that its men are not medical men, and dentistry is a separate profession in practice if not in theory.

The added knowledge, the broader outlook on pathology and therapeutics, the larger privileges in surgery would have been of immense value to the practicing dentist.

You may say that dentistry is recognized as a specialty in medicine. It is so declared by every code of *dental* ethics. *We* recognize it all right, but you will agree with me that it receives scant acknowledgment from the medical profession.

The ophthalmologist, aurist, and many other "ists" have full recognition, but when was the dental fraternity honored with an invitation to enjoy the sessions of a medical convention, much as they might add to the interest and be benefited thereby? The reason is not far away. Dentistry is looked upon as a distinct affair, with no practical connecting links.

And why, gentlemen, does not the degree of D. D. S., or D. M. D., or L. D. S., carry as much prestige as M. D.?

It is somewhat owing to ignorance of the scope of the curriculum of the Dental College of to-day, but largely because the standard of matriculation and the college course of the dental student has not been equal to that required from the student of medicine.

The Dental College has not required as high a standard of matriculation; the professional course is not recognized as being as thorough nor as long as that of the well-conducted Medical College; also because of having a separate degree, dentistry is naturally placed by itself and takes a different level.

We claim that the specialty of dental surgery should stand by the side of all other specialities in medicine, untrammelled, and receive full recognition as an integral part of the great medical whole.

This would have been the case had the Dental Colleges been post graduate schools for our specialty. Dentistry would then have taken its proper and recognized place years ago, and all the bitter fight would have been saved.

We venture to hope that the evolution is in progress; indeed, there are signs of it on evey hand.

The dental degree should by no means be abolished. The specialty has so much that is distinct and unique, that it well merits special recognition. It has a pathology, therapeutics, and Materia Medica of its own. Its manipulations are complicated and delicate. No specialty *needs* so much preparation as ours.

The medical man does know something of the eye, ear, throat, and any of the other specialties, but he knows *nothing* of dentistry. "Where is the medical man who is not a dentist who can stand at a dental chair and operate five minutes with credit to himself or comfort to his patient?

There is no department of surgery where more delicacy of touch is needed; none where such a variety of appliances are necessary; none where more scientific diagnosis and treatment are required.

The whole outfit of the general surgeon is not as complicated and elaborate as that of the dental surgeon. A special degree should certainly be retained, but I do think it should express the full size of the field of our specialty, as is generally understood by the terms Stomatology or Oral Surgery.

If I am correctly interpreting the signs of the times, the feeling is growing that the two degrees belong together. The public demands oral surgery from the dentist now, and may (with the number rapidly increasing) feel that they need and must have the general medical and surgical training, and the privileges that accompany it.

The professors in the Dental Colleges feel the need, and a majority of them write M. D., as well as D. D. S. The Dental Colleges feel the need of close contact, and the best schools of dentistry to day are either departments of universities or are affiliated with medical colleges, and having arrangements whereby both degrees may be secured at a minimum loss of time.

At the last meeting of the National Board of Dental Faculties, a resolution was offered and tabled for discussion next year, which proposes to add another year to the Dental College course. This is adding at the wrong place.

As far as I have been able to gather, and I have taken some trouble to be informed, the idea of a longer course is not so much to add to the lectures on special dental subjects as to give opportunity for those studies that are included in general medicine and surgery. Why then, if this be so, and the time is to be spent pursuing these subjects, should not the dental student of the future have the the recognition that comes from a medical degree? A resolution looking toward a medical degree as preliminary and necessary for the student of dentistry would be more to the point, and much nearer the ideal.

The public are demanding more and more from the dental surgeon. The profession is looking in the direction indicated in this paper. Higher qualifications are demanded from students every year. State and Provincial Boards are raising their standards. As combined in the National Board of Dental Examiners, they are stimulating the colleges to better things, and to-day the general trend is turning in the direction of medical training as a preparation for the specialty of oral surgery.

This paper is short. It has been my purpose to only touch the subject in order to introduce it and give time for a discussion.—*Dominion Dental Journal.*

